# Leptospirosis in Ruminants in Yogyakarta, Indonesia: A Serological Survey with Mixed Methods to Identify Risk Factors

**DOI:** 10.3390/tropicalmed6020084

**Published:** 2021-05-20

**Authors:** Dyah Ayu Widiasih, Johanna Frida Lindahl, Wayan T. Artama, Adi Heru Sutomo, Pande Made Kutanegara, Guntari Titik Mulyani, Estu Widodo, Tjut Sugandawaty Djohan, Fred Unger

**Affiliations:** 1 Department of Veterinary Public Health, Faculty of Veterinary Medicine, Universitas Gadjah Mada, Yogyakarta 55281, Indonesia; dyahaw@ugm.ac.id; 2 One Health Collaborating Center-Universitas Gadjah Mada (OHCC-UGM), Yogyakarta 55281, Indonesia; artama@ugm.ac.id (W.T.A.); adi.heru.sutomo@ugm.ac.id (A.H.S.); kutanegara@ugm.ac.id (P.M.K.); tjutdjohan@ugm.ac.id (T.S.D.); 3 International Livestock Research Institute (ILRI), Hanoi 100000, Vietnam; f.unger@cgiar.org; 4 Department of Medical Biochemistry and Microbiology, Uppsala University, 75123 Uppsala, Sweden; 5 Department of Clinical Sciences, Swedish University of Agricultural Sciences, 75007 Uppsala, Sweden; 6 Department of Biochemistry, Faculty of Veterinary Medicine, Universitas Gadjah Mada, Yogyakarta 55281, Indonesia; 7 Department of Public Health, Faculty of Medicine, Public Health and Nursing, Universitas Gadjah Mada, Yogyakarta 55281, Indonesia; 8 Department of Anthropology, Faculty of Cultural Science, Universitas Gadjah Mada, Yogyakarta 55281, Indonesia; 9 Department of Internal Medicine, Faculty of Veterinary Medicine, Universitas Gadjah Mada, Yogyakarta 55281, Indonesia; guntari@ugm.ac.id; 10 Department of Agriculture and Food, Kulon Progo District, Ministry of Agriculutre Indonesia, Yogyakarta 55652, Indonesia; trontong_estu@yahoo.com; 11 Ecology Laboratory, Faculty of Biology, Universitas Gadjah Mada, Yogyakarta 55281, Indonesia

**Keywords:** *Leptospira*, neglected tropical disease, zoonosis, livestock production, Ecohealth, Southeast Asia

## Abstract

Leptospirosis is a zoonotic disease occurring worldwide with reproductive symptoms and production losses in livestock, while humans can suffer fatal renal failure. In Yogyakarta Special Province, Indonesia, there have been several outbreaks with high case fatality, demonstrating the public health importance, but there is limited understanding of the epidemiology. This study used an EcoHealth approach to ensure transdisciplinarity and community participation. Seroprevalence of *Leptospira* in animals was studied between October 2011 and May 2013 in 15 villages. Serum samples from 1404 cattle and 60 small ruminants were screened by a Microscopic Agglutination Test (MAT), first in pools, and then the individual positive samples were identified. Focus group discussions including farmers, village officials, and official stakeholders were used to explore knowledge and behavior of zoonotic diseases, particularly leptospirosis. Two small ruminants were seropositive for *Leptospira icterohemorrhagiae*. From the cattle, 3.7% were seropositive, and the most common serovars were *Leptospira hardjo*, followed by *L. icterohemorrhagiae*. Out of all farms, 5.6% had at least one positive cattle. Risk factor analyses showed that the risk of the farm being seropositive increased if the farmer used water from an open source, or if farming was not the main occupation. This study showed the presence of *Leptospira* spp. in ruminants in Yogyakarta and identified use of open water as a risk factor for the livestock. We also observed that the knowledge related to leptospirosis was low, and risky farm management practices were commonly employed.

## 1. Introduction

Leptospirosis is caused by spirochetal bacteria of the genus *Leptospira*. It is a zoonotic disease occurring worldwide, and most parts of Asia are considered likely to have high incidence of human leptospirosis, even though data are missing for most countries [1]. The disease causes reproductive problems and production losses in livestock, while mortality in humans is usually related to renal failure [1,2]. Leptospirosis is most common in tropical and subtropical areas with high rainfall and humidity and is often considered a rural disease. Under conditions where communities and related health services pay little attention to environmental health, *Leptospira* spp. can easily circulate undetected [3]. The observed re-emergence of leptospirosis in Indonesia is thought to be caused by multiple factors and highly linked to environmental conditions such as flooding, high density of potential hosts, and socioeconomic factors [4]. Thus, leptospirosis may pose a severe and strongly underestimated health problem in Indonesia [5,6]. National data for the case fatality of human leptospirosis indicated an average of 7.1% (range 2.5%–16.5%) for the time period from 2003 until 2007 [7]. In a survey of more than 1400 febrile cases, 3% were found to be caused by *Leptospira*, and the authors concluded that limited diagnostic capabilities as well as often non-typical cases complicate diagnosis [8]. There has been a growing interest in studying leptospirosis in Indonesia, but this has focused on human incidence and rodents, and less has been done to estimate the occurrence in the ruminant population.

For a more effective response, the epidemiology of leptospirosis and related health impacts needs to be better understood. Leptospirosis has been ranked twelfth in importance among animal diseases by the Ministry of Agriculture of Indonesia. Classical sector-specific approaches have failed to be successful in control and prevention, and therefore it is important to adopt a transdisciplinary and participatory approach to understanding and mitigating leptospirosis in Indonesia. Based on reports of Municipal Health Authorities, outbreaks of human leptospirosis in Yogyakarta Special Province have been increasing since 2011, particularly in Bantul and Kulon Progo District in the southern areas of Yogyakarta Special Province. In Kulon Progo District, Yogyakarta Special Province, the reported case fatality rates were 5.8% for 2011, 7.1% for 2012, and 33.3% for 2013 [9,10]. This project was implemented as part of a larger EcoHealth initiative to create the necessary linkages between stakeholders, policy makers, and researchers [11]. Similar to the One Health approach that connects human and animal health with environmental health and drivers, EcoHealth is a transdisciplinary approach that aims at reaching sustainable improvements in animal, human, and ecosystem health, and encourages participation of different stakeholders [11,12,13].

The objective of this study was to explore the knowledge and behavior of groups at risk and reveal potential risk factors for leptospirosis in humans and animals by using qualitative (focus group discussions (FGDs)) and quantitative (questionnaires) methods for data collection, and serum samples from livestock were used to estimate the seroprevalence.

## 2. Materials and Methods

This project was conducted between October 2011 and May 2013 and used a transdisciplinary approach involving physicians, veterinarians, ecologists, socioeconomic experts, a demographist, an anthropologist, and stakeholders in the design and implementation.

### 2.1. Farm Survey

Farms were visited during the early wet season between November and December 2011. Data on human leptospirosis incidence in 2010 were collected from the Municipal Health Services, and a selection of eight sub-districts in Kulon Progo District, Yogyakarta Province, were purposively selected to represent different levels of human incidence. Fifteen villages were selected from these sub-districts, using proportional random sampling based on cattle population estimates from the local authorities. All farmers keeping livestock (cattle or small ruminants) in the villages were visited, and blood samples were collected from all cattle that the farmer would allow sampling from, with between 1 and 12 cattle being sampled per household. In addition, four small ruminants were sampled per village. Following this, 1464 ruminants (1404 cattle and 60 small ruminants) were sampled from 775 villages (Table 1).

Data on farmer demographics, farm management, and animals for all the sampled livestock were collected. Further, farmer knowledge about leptospirosis was assessed using a pre-tested questionnaire. The local name *Penyakit Kencing Tikus,* or “rat’s urine disease”, was used for leptospirosis.

### 2.2. Serological Analyses

Serum was separated by centrifugation and frozen, and before analysis, samples were thawed and pooled in pools of 2 or 3. The laboratory testing was done at the Central Veterinary Research laboratory, Bogor, West Java. Analyses were done using the Microscopic Agglutination Test (MAT) to identify anti-*Leptospira* antibodies at Central Veterinary Research, Bogor, West Java. This serological test used 14 serovars from 14 serogroups; Serogroup Icterohemorrhagiae (serovar Icterohemorrhagiae), Javanica (serovar Javanica), Celledoni (serovar Celledoni), Canicola (serovar Canicola), Ballum (serovar Ballum), Pyrogenes (serovar Pyrogenes), Cynopteri (serovar Cynopteri), Autumnalis (serovar Rachmati), Australis (serovar Australis), Pomona (serovar Pomona), Grippotyphosa (serovar Grippotyphosa), Sejroe (serovar Hardjo), Bataviae (serovar Bataviae), Tarrassovi (serovar Tarrassovi). The procedures of the MAT followed the guidelines provided by the World Health Organization [14]. When a serum pool tested positive, the test was repeated for each individual sample, to identify the infected animal. A titer of 1:100 or above was considered positive, the most common cut-off according to a meta-analysis of serological surveys in animals in Southeast Asia [15].

### 2.3. Risk Factor Analyses

Data were entered in Excel and data analyses were conducted in STATA 14.0 (StataCorp LP, College Station, TX, USA), using Chi^2^ or Fisher’s Exact test for univariable analyses. For risk factor analyses, small ruminants were removed. Univariable associations with serological status were tested for the following variables: cattle age and breed, farmer’s age, occupation and education, water sources, floor composition, cleanliness, presence of rats, and previous sick cows or abortions. Logistic regression (xtmelogit) was used for the multivariable models with serological status at farm level as the dependent variable, independent variables identified in the univariable analyses (*p* < 0.2), and village included as a random effect. Manual backward elimination of variables was done to optimize the model, considering *p* < 0.05 significant and keeping factors with an effect on other variable estimates of more than 20% as a confounder. Multivariable analyses for animal level risk factors were not performed.

### 2.4. Participatory Methods

Six FGDs were conducted in April–May 2013 in three sub-districts categorized according to unpublished reports of human leptospirosis into low, medium and high-risk sub-districts. In each of these locations two FGDs were organized. FGDs included farmers, village officials, and stakeholders from Municipal Health Services and Livestock Services (Table 2) to explore potential risk factors and collect background information regarding leptospirosis. More specifically, this included knowledge of zoonotic diseases focusing on leptospirosis, human behavior related to control, prevention, and existing policies. The duration of the FGD was usually 1.5–2 h, with 10 to 15 people in each group.

## 3. Results

### 3.1. Farm Survey

In total, 775 households were included in the serological survey. Of the 760 respondents that answered the question on education, 51.3% had no education higher than primary school, and 27.1% had passed high school. The average age of participants was 51.8 years, and the age ranged from 25 to 85 years. For 682 participants (89.0%) farming was the primary occupation, and the others were either entrepreneurs or civil servants. Using the local name “rat’s urine disease”, 466 (62.4%) answered they had never heard of that disease. Of those that had heard about it, 43.0% had heard it through mass media, and 56.0% heard about it through extension services. A total of 146 out of the 250 that had heard about leptospirosis also stated that they believed that either themselves or someone in the neighborhood had suffered from the disease. Only two farms reported grazing the cattle at pasture, and similarly two farms reported that they used the cattle as draft power.

### 3.2. Serological Analyses

**Figure 1:**
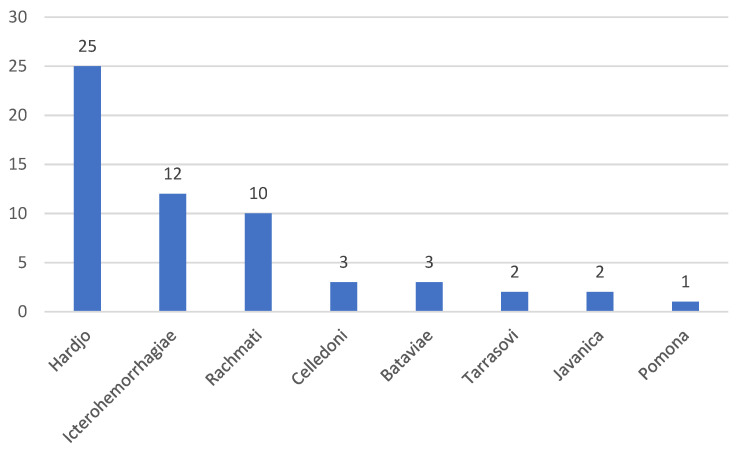
Number of positive cattle for each *Leptospira* serovar.

In total, 1404 cattle were tested using the MAT, and 52 of these (3.7%, 95% confidence interval (CI) 2.8–4.8%) were positive for at least one serovar. Only 60 small ruminants were sampled, and no distinction was made between sheep and goats. Out of these, two were positive (3.3%, 95% CI 0.4–11.5%) and both were positive for the serovar Icterohemorrhagiae. Among cattle, the most common serovar was Hardjo, followed by Icterohemorrhagiae (Figure 1). Three cattle had a serological response to more than one serovar; one cow was positive for the serovars Icterohemorrhagiae, Javanica, and Celledoni with equally high titers (1:100 to each), while two cows were positive for Hardjo and Rachmati, but 16 times higher titer against Hardjo. On a herd level, 43 farms had at least one animal (two with small ruminants, and 41 with cattle) positive (5.6%, 95% CI 4.0–7.4%). The herd prevalence, including only farms with cows, was 5.4% (95%, CI 3.9–7.3%).

### 3.3. Risk Factor Analyses

There were significant differences between villages, with two villages having more than 15% positive animals, and other villages having no positive animals (Table 1).

#### 3.3.1. Farm Level Analyses

There were no significant differences in the proportion of farms that had experienced abortion between positive and negative farms (48.8% and 55.7%, respectively) or in the proportion of farms that had sick cattle the last year (36.6% and 26.0%, respectively).

Significantly (*p* = 0.035) more farms were seropositive when the farmer stated that the main occupation was not farming (11.0% compared to 4.8% in farmer households). Providing water from an open water source was also significantly associated with higher herd prevalence (14.1%, *p* = 0.003), compared to well or tap water (4.3%). In 722 farms observations on the floors were made; 27.3% had a soil floor, and 72.7% had permanent floors. However, this was not associated with herd positivity. Among the farms judged to have clean floors, 7.5% were positive, compared to 3.6% in dirty stables (*p* = 0.020). Water source and cleanliness were, however, correlated, with 56.5% of farms with open water sources being clean compared to 31.9% among other farms (*p* < 0.001). The presence of rats, categorized as no rats observed, fewer than 5, or more than 5, was not associated with positivity, either in the fields, in the feed storage, or among the livestock. Neither the age of the farmer nor level of education was associated with the farm having seropositive cattle.

The proportion of participants working mainly as farmers as well as the proportion of farms using open water sources varied significantly among the different villages. Thus, the final multivariable model only included water source and occupation, showing that both were associated with risk for leptospirosis (Table 3).

#### 3.3.2. Animal Level Analyses

Most cattle were female (87.8%), and the most common breed sampled was Simmental (46.1%), followed by Limousine (18.1%). For individual cattle, breed and sex were not significantly associated with the animals being seropositive. In total, 2.6% of cows had aborted, but no aborting cow was seropositive for *Leptospira*.

### 3.4. Participatory Methods

The number of participants for the six FGDs organized reached 70 in total. The majority were farmers (25%), followed by cattle farmer leaders (15%), livestock service officers (13%), heads of villages (10%), and municipal health officers (7%). The majority of participants (81.5%) were male. Most of the participants were knowledgeable about common zoonoses such as rabies and avian influenza, but only very few had heard about leptospirosis. In each focus group, only one person had heard about leptospirosis, and the most common knowledge about leptospirosis was that it was caused by rodents, but they did not know that other animals, such as livestock or even humans, could spread the disease through their urine. They also reported that some people from the neighborhood had been infected by the “rat urine disease”, *Penyakit Tikus*, including some who died, and this was most often leptospirosis. Risk behaviors reported included not using boots in the rice field and washing hands with rice field water. The knowledge on leptospirosis transmission, if there was any, included that it was mainly spread by rodent urine, rice field water, contact with the infected animal, but participants were still confused about whether all species of rodents could spread it or not. According to FGD participants, rodents were often found on the roof of the house, in the backyard, in piles of grain in the warehouse, and in the rice field. Species of rats seen and identified by participants varied from *Rattus argentiventer* (local name *wirog*), *Rattus rattus* (*pithi*), *Rattus norvegicus* (*clurut*), and *Rattus tiomanicus* (reported to be mostly in the trees). They are all considered local rats, with mostly *pithi* found in homes. Participants reported that natural predators of rodents, such as snakes, *garangan* (*Herpestes javanicus,* also called cerpelai/Javan mongoose), or owls, were commonly captured to sell or killed, which could cause increasing numbers of rodents. Some participants stated that chemicals were used frequently to fight rats. Other ways of rodent control were killing, burning, and being burned by traditional fireworks (bombing). Farmers also stated in FGDs that when rats were killed by poison in the rice field, other rat colonies soon emerged.

## 4. Discussion

This study shows that leptospiral infection occurs in ruminants in Yogyakarta, Indonesia. The serovar *L. hardjo* was most common, followed by *L.* *icterohemorrhagiae. Leptospira hardjo* is known to be host-specific for cattle and the most important cause of leptospirosis in cattle in the United States [16], and is a reported cause of abortions in many other countries, including Indonesia, Iran, USA, Canada, Turkey, Brazil, and Mexico [17,18,19,20,21,22]. This study found a seroprevalence in cattle of 3.7%, which is considerably lower than previous studies. However, this study was not conducted on a completely random sample of animals and farms, and was thus not representative of a larger area, but mainly serves to show the serovars circulating and the risk practices in the risk areas. Some earlier studies in Indonesia have reported much higher prevalence than our present study. Scott et al. [23] reported up to 37% of dairy cattle tested to be positive for *Leptospira* serovar Hardjo and Tarassovi for various regions of Indonesia, but also found 0% prevalence in Bali and 3.5% in North Sumatra, indicating a high variability. Another study indicated 16.7% for serovar Hardjo for Yogyakarta Special Province [24]. Diarmita et al. [25] reported 33% prevalence of *Leptospira* on the Lombok Island. A much smaller study in Yogyakarta found two out of ten cattle seropositive for *Leptospira* [26]. The lower prevalence in our study for *L. hardjo* might be related to factors such as the production system. Grazing has been reported to be an important risk factor for leptospirosis in Brazil [27], but only two farms included in this survey were grazing their cattle. Previous research in Yogyakarta stated that the presence of rodents around the house increased the incidence of human leptospirosis by 7.4 times [28], but there was no evidence in this study that rodents increased the risk for a farm to have infected cattle. Rodents were assessed by observation at the time of interviewing, and it could be that this method was too insensitive to accurately measure this risk factor. Another explanation could be that there are very few rodents around cattle stables in the area due to the chemicals used to control the rodent population. This could help to control the prevalence of leptospirosis. Pooling serum for MAT analysis may also decrease sensitivity, which means the level of seropositivity shown here could be an underestimation.

The FGDs identified common rodents around the farms, and although rodents were not tested here, studies in other parts of Southeast Asia have found both different rodent and insectivore species seropositive, with a meta-analysis indicating a likely overall seroprevalence of 18% [15]. Identifying rodent reservoirs in this part of Indonesia still needs to be done.

The lack of knowledge of zoonosis among farmers and other stakeholders could be a predisposition factor that influences the prevalence of leptospirosis in humans and animals. Interestingly, this study found that the risks for a farm to be positive were higher if farming was not the main activity. This may indicate that a dedicated professional farmer may be better at protecting the animals by risk mitigation practices not captured in this survey.

One major risk factor identified in this study was the use of open water sources, river, ponds, etc., which is consistent with findings of water being a major risk for *Leptospira* transmission [4,29]. We also found a link with cleanliness, which may be because farms using open water sources are using more water to clean the farm and thereby potentially spreading leptospires inadvertently. In this study, most farms kept their livestock in zero-grazing systems, which is likely to increase further as production intensifies. Inadequate infrastructure may mean that many farmers need to continue using open water sources. Our study also reported that the farmers connected more rats with the reduction of predators such as owls. Owl breeding can be an effective way of controlling rodents, as has been shown in other parts of Indonesia [30].

The qualitative data collected in our study demonstrated that most farmers have very little knowledge about leptospirosis. The limited knowledge was restricted to the disease being caused by rodents, and they did not know that other livestock, and even infected humans, could spread the disease through their urine. The focus groups reported that the majority of farmers followed risky habits, such as not using boots in the rice field and washing hands with rice field water. At the time of our study, some education had been delivered by Municipal Health Authorities, focusing in particular on people in areas with high human incidence, which may have contributed to an improved understanding of leptospirosis and changed behavior, but knowledge was still very low. Despite these extension efforts to control leptospirosis, jointly carried out by personnel from Municipal Health Services and Livestock Services, with emphasis on community education of zoonotic diseases, our results showed that knowledge of risk groups is still insufficient. Poor knowledge was earlier found to be associated with a higher risk of leptospirosis in humans, pointing to the importance of community awareness programs [31]. Our study results suggest strengthening and further expanding ongoing efforts, and also provide more evidence on risk factors that these community awareness programs can focus on.

Although the burden of human leptospirosis is likely high in all Southeast Asia, it has been difficult to make estimates due to the lack of official data, and this is also true for Indonesia [1]. However, as with other zoonotic diseases, farmers also struggle with the associated production losses from the diseases, which can have substantial impacts on their livelihood [32].

## 5. Conclusions

This study confirmed the circulation of *Leptospira* among ruminants in Yogyakarta and that the use of open water was a contributing risk factor. Moreover, it confirmed that knowledge of risk groups related to leptospirosis was, in general, low and risky habits were common. From the results of this study we see the need for more education about leptospirosis, and also new scopes of research to study rodent populations and the infection in them, as well as the need to look more into the variability of prevalence among different areas and the causes. This study was successfully designed and implemented by a transdisciplinary research team consisting of experts from various faculties of the university and can act as a model for future One Health and EcoHealth projects and initiatives.

## Figures and Tables

**Table 1:** Number of sampled households and animals, as well as the proportion using open water sources, and the seropositivity for leptospirosis at household and animal level in Yogyakarta Special Province, Indonesia.

Sub-District	Risk *	Village	Open Water Source	Cattle Sampled (Seropositives, %)		Small Ruminants Sampled (Seropositives, %)	
				Households	Animals	Households	Animals
Girimulyo	High	1	59.3%	65 (9, 13.9%)	132 (10, 7.6%)	1 (0, 0%)	4 (0, 0%)
Kalibawang		2	55.6%	18 (4, 22.2%)	23 (5, 21.7%)	2 (0, 0%)	4 (0, 0%)
Lendah		3	0%	0 (0, 0%)	0 (0, 0%)	2 (0, 0%)	4 (0, 0%)
Lendah		4	0%	129 (6, 4.7%)	205 (6, 2.9%)	1 (0, 0%)	4 (0, 0%)
Lendah		5	0%	51 (0, 0%)	74 (0, 0%)	2 (0, 0%)	4 (0, 0%)
Nanggulan		6	0%	25 (1, 4%)	46 (1, 2.2%)	4 (0, 0%)	4 (0, 0%)
Panjatan	Medium	7	0%	36 (1, 2.8%)	79 (1, 1.3%)	2 (0, 0%)	4 (0, 0%)
Panjatan	Medium	8	0%	55 (2, 3.6%)	118 (2, 1.7%)	3 (1, 33%)	4 (1, 25%)
Pengasih	Low	9	97.5%	25 (0, 0%)	37 (0, 0%)	2 (0, 0%)	4 (0, 0%)
Pengasih	Low	10	0%	109 (3, 2.7%)	179 (4, 2.2%)	2 (0, 0%)	4 (0, 0%)
Samigaluh		11	74.2%	16 (4, 25%)	27 (4, 14.8%)	2 (1, 50%)	4 (1, 25%)
Wates		12	0%	76 (1, 1.3%)	137(1, 0.7%)	2 (0, 0%)	4 (0, 0%)
Wates		13	2.4%	52 (0, 0%)	123 (0, 0%)	2 (0, 0%)	4 (0, 0%)
Wates		14	0%	20 (7, 35%)	61 (11, 18%)	2 (0, 0%)	4 (0, 0%)
Wates		15	0%	76 (3, 4.0%)	163 (7, 4.3%)	2 (0, 0%)	4 (0, 0%)
		Total	9.2%	757 (41, 5.4%)	1404 (52, 3.7%)	31 (2, 6.4%)	60 (2, 3.3%)

* Expected risk for animal leptospirosis in Kulon Progo District, Yogyakarta Special Province, based on data from human leptospirosis.

**Table 2:** Overview of the participants in focus group discussions (FGDs) conducted in low, medium and high-risk sub-districts.

Sub-District	Low Pengasih		Medium Panjatan		High Girimulyo	
	FGD 1	FGD 2	FGD 3	FGD 4	FGD 5	FGD 6
Farmer	5	4	3	5	4	4
Head of village	2	1	3	1	2	1
Cattle farmer leader	3	3	2	2	2	3
Municipal health officer	1	1	1	1	2	1
Livestock services	1	1	4	1	5	1
Total	12	10	13	10	15	10

**Table 3:** Results from a multivariable logistic model on risk factors for a herd having at least one animal positive for *Leptospira* antibodies.

	Odds Ratio	z	*p*-Value	95% Confidence Interval
Having another primary occupation	3.0	2.7	0.007	1.4–6.8
Using open water source compared to well or tap water	4.0	3.4	0.001	1.8–9.1
